# Comparison of Aerodynamic and Elastic Properties in Tissue and Synthetic Models of Vocal Fold Vibrations

**DOI:** 10.3390/bioengineering11080834

**Published:** 2024-08-15

**Authors:** Jacob Michaud-Dorko, Charles Farbos de Luzan, Gregory R. Dion, Ephraim Gutmark, Liran Oren

**Affiliations:** 1 Department of Biomedical Engineering, University of Cincinnati, 665 Baldwin Hall, Cincinnati, OH 45221-0070, USA; diongy@ucmail.uc.edu (G.R.D.); orenl@ucmail.uc.edu (L.O.); 2 Department of Otolaryngology-Head and Neck Surgery, University of Cincinnati, 231 Albert Sabin Way, Cincinnati, OH 45267-0528, USA; farboscs@ucmail.uc.edu (C.F.d.L.); gutmarej@ucmail.uc.edu (E.G.); 3 Department of Aerospace Engineering, University of Cincinnati, 799 Rhodes Hall, Cincinnati, OH 45221-0070, USA

**Keywords:** phonation, vocal fold models, vertical stiffness gradient, divergence angle, flow separation vortices

## Abstract

Three laryngeal models were used to investigate the aerodynamic and elastic properties of vocal fold vibration: cadaveric human, excised canine, and synthetic silicone vocal folds. The aim was to compare the characteristics of these models to enhance our understanding of phonatory mechanisms. Flow and medial glottal wall geometry were acquired via particle image velocimetry. Elastic properties were assessed from force–displacement tests. Relatively, the human larynges had higher fundamental frequency values, while canine and synthetic models exhibited greater flow rates. Canine models demonstrated the highest divergence angles and vertical stiffness gradients followed by the human model, both displaying flow separation vortices during closing. Synthetic models, whose advantage is their accessibility and repeatability, displayed the lowest glottal divergence angles and total circulation values compared to tissue models with no flow separation vortices. The elasticity tests revealed that tissue models showed significant hysteresis and vertical stiffness gradients, unlike the synthetic models. These results underscore the importance of model selection based on specific research needs and highlight the potential of canine and synthetic models for controlled experimental studies in phonation.

## 1. Introduction

The combination of three significant components causes the production of voice: the aerodynamic pressure from the lungs, the sound source from the vocal folds, and the vocal tract resonator, commonly referred to as the filter [1,2]. The classic source filter theory states that the source of sound is produced at the glottis by flow modulations, referred to as phonation [3]. During phonation, the vocal folds are excited into a flow-induced self-oscillatory state. Vocal fold vibration converts a steady stream of air supplied by the lungs into a pulsating stream of airflow. The vibration changes the volumetric airflow (Q) that passes through the glottis as a function of time (dQ/dt). The volumetric airflow is known as the glottal flow waveform. It can be separated into three phases: opening, closing, and closed. During this flow-induced self-oscillatory state, the vocal folds alternate between a convergent (during the opening phases) and divergent (during the closing phases) glottal shape. The greatest rate of airflow change occurs during the closing part of the glottal flow cycle. It is referred to as the maximum flow declination rate (MFDR) and is of clinical importance because it presents information on radiated sound pressure levels (SPLs) and vocal efficiency (VE) during speech [4].

The convergent–divergent shape during phonation occurs because of a vertical stiffness gradient (VSG), which ultimately effects and controls the fluid dynamics within the glottis. The intraglottal flow will remain attached to the glottal wall during the opening phases as the glottis takes on the shape of a convergent nozzle. However, the flow will separate from the vocal fold wall when the glottis forms a divergent nozzle during the closing phases. Intraglottal flow separation during closing has been observed in computational and experimental models [5,6,7,8]. Flow separation produces vortices on one or both sides of the main jet, defined as areas of rotational motion in the upper part of the glottis. The divergent duct creates an adverse pressure gradient, which leads to flow separation along the glottal wall, required to form flow separation vortices (FSVs). Larger divergence angles produce more significant adverse pressure gradients, resulting in stronger and larger FSVs. It is commonly understood that the flow will typically separate when the nozzle half angle exceeds approximately 15°, which means that the nozzle may experience internal flow losses caused by flow separation [9,10]. These FSVs are different to shedding vortices because they remain stationary within the glottis during closing, where shedding vortices propagate downstream in the shear layer of the jet, known as Kelvin–Helmholtz vortices.

During phonation, the intraglottal pressure distribution determines the forces applied to the glottal wall. FSVs create negative gauge pressures that reduce the fluid resistance to the elastic forces that close the glottis [5]. These negative gauge pressures have been correlated with increased closing speeds during the glottal cycle (i.e., glottal flow skewing towards closing) [8], where the skewness index (SI) is defined as the ratio of the duration of the opening phase over the duration of the closing phase of the glottal cycle [11]. In addition, FSVs have also been correlated with a higher SPL and VE [12].

There is a phase delay between the inferior and superior edges of the glottis (i.e., vocal fold), with the inferior edge opening and closing before the superior edge. This phase delay is known as the vertical phase delay and is the result of a decreasing VSG along the height of the vocal fold, from the inferior to superior edge. A greater vertical phase delay is associated with greater divergence angles, increased acoustic intensity, VE, cepstral peak prominence (CPP), and stronger FSVs [13]. Where acoustic intensity is a measurement of the SPL, VE is defined as the acoustic power over the aerodynamic power [14], and CPP is an acoustic measure to determine dysphonia severity [15,16].

A computational study utilizing a two-mass model showed that the VSG is necessary for physiological phonation characteristics [17]. Experiments have also shown the presence of a VSG through mechanical testing with cadaveric human and excised canine larynges [18,19,20,21]. It was shown that, at low strains or displacements, the superior edge is about as stiff as the inferior edge. However, as the displacement increases, the inferior edge becomes much stiffer than the superior edge. Therefore, at similar intraglottal pressures, the superior aspect of the glottis will displace more laterally, which translates to a greater maximum divergence angle during phonation [22]. When the VSG was decreased in an excised canine larynx model, the maximum divergence angle decreased, which reduced or eliminated the FSVs, resulting in lower VE [13]. Therefore, to increase VE, a larger divergence angle is needed to increase FSVs, which is caused by a greater vertical phase delay, which is driven by a greater VSG.

Finding an ideal model of the human larynx is important for studying the underlying mechanism of human phonation. The validity of data obtained using vocal folds from a cadaveric human larynx can be limited because these larynges are typically obtained seven days postmortem. Additionally, most cadaveric human larynges are from older individuals, where the premortem atrophy of the vocal folds’ tissue is common, making the excised cadaveric human larynx harder to phonate.

Vocal folds from an excised canine larynx are one of the most common tissue models used to study phonatory processes. Despite some significant morphological differences, canine vocal folds have been espoused as a reasonable approximation to human folds because of their geometrical similarity and comparable acoustic output in terms of fundamental frequency (*f*_o_) and amplitude of vibration [23,24]. The cartilage and muscles in a canine larynx have dimensions and orientations similar to a human larynx. However, the canine larynx is approximately 40% larger than the human larynx and requires higher phonation pressures because its vocal ligament is stiffer. Fortunately, similar stress–strain curves have been observed, which show how both tissue models vocal folds possess nonlinear viscoelastic behaviors [25].

Synthetic models of the vocal folds were developed to overcome some of the tissue models’ limitations, such as availability, cost, and the ability to obtain repetitive data. The synthetic models are made of silicone and intended to mimic the geometry and material properties of human vocal folds [26]. Relative to the tissue models, synthetic models are more readily accessible and can sustain vibration for more extended periods. These synthetic models also allow for material properties and model geometries to be parametrically varied to observe the effects of these changes on the models’ flow-induced vibratory responses [27]. Synthetic models of vocal folds have been used to determine contact pressure during phonation [28], to study asymmetries during phonation [29,30,31], and the impact of subglottal stenosis constrictions on phonation [32].

The main aim of this study is to compare key characteristics of tissue (human and canine) and synthetic vocal fold models during vibration. Specifically, it focuses on glottal wall geometry and intraglottal aerodynamics during phonation in these three models. This study aims to compare the biomechanical properties of each model to explain the cause of the vertical phase delay and divergence angle seen at the MFDR phase. To date, the intraglottal flow field in cadaveric human laryngeal tissue and in the multi-layered synthetic epithelium (EPI) vocal fold model created by Murray and Thomson [26] has not been documented in the literature. The overall goal of this study is to characterize the intraglottal flow field and biomechanical properties for each model, providing computationalists with data to validate their models. Furthermore, it aims to offer researchers a potential guideline for which model is most appropriate for their studies, given the advantages and limitations of each model in terms of glottal wall geometry, aerodynamic characteristics, acoustic output, and biomechanical properties.

The results of this study will include an analysis of the glottal width and flow waveform in the mid-coronal plane at the superior aspect for all vocal fold models. The intraglottal flow field at the MFDR phase will then be presented to investigate intraglottal flow separation, divergence angle, vorticity, and total circulation. Finally, the study will analyze the stress–strain characteristics and Young’s modulus along the vertical height of the vocal fold in each model to compare their biomechanical properties. The intent is to demonstrate the importance of the VSG and its correlation with greater divergence angles at the MFDR phase, increased circulation, higher VE, and glottal skewing towards closing (SI > 1).

## 2. Materials and Methods

### 2.1. Models Preparation

Three cadaveric human larynges (HL1–HL3) were harvested postmortem, and six canine larynges (CL1–CL6) were harvested immediately after the animals were euthanized. All cartilage and soft tissue above the vocal folds were removed to gain optical access to the vocal folds during phonation. The larynges were kept in saline and stored at 3.3 °C in a refrigerator overnight between tests. Six synthetic silicone models (SL1–SL6) were fabricated following the instructions for a multi-layer, self-oscillating, EPI vocal fold [26]. The ligament thread was omitted in these models to enable obtain mid-coronal glottal widths (i.e., 2–3 mm displacement distance between the folds) and vibration frequencies above 100 Hz [27].

### 2.2. Experimental Setup for Aerodynamic Measurements

**Figure 1:**
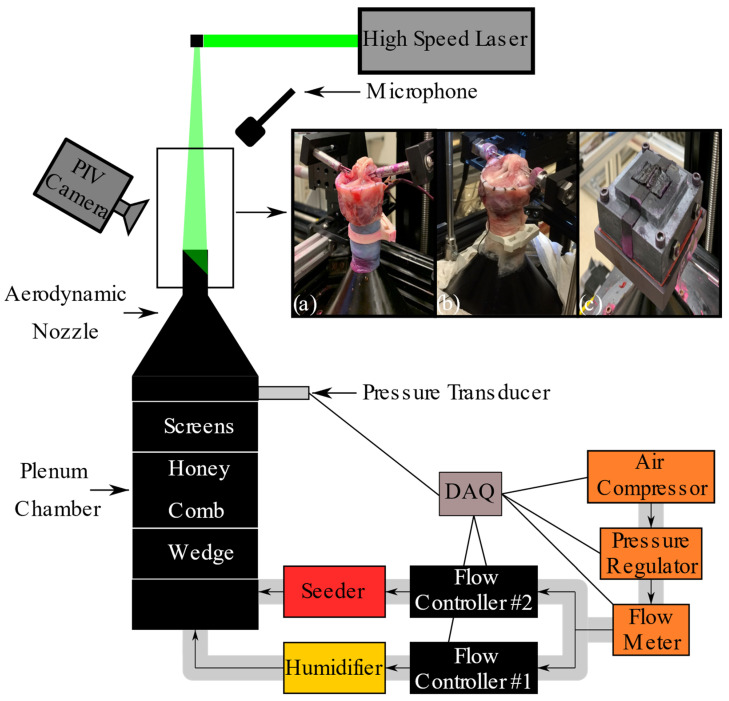
Schematic of the experimental setup used to capture the intraglottal flow field and glottal wall geometry using particle image velocimetry (PIV). (**a**) Cadaveric human larynx, (**b**) excised canine larynx, and (**c**) synthetic larynx, all mounted on an aerodynamic nozzle.

To measure the glottal geometry and flow field, the models were mounted on an aerodynamic nozzle connected to a cylindrical settling chamber (101 mm ID, 250 mm long), which conditioned the airflow before it entered the glottis (Figure 1). The chamber was equipped with a perforated wedge, honeycomb, and screens to ensure proper airflow conditioning. The airflow transitioned from the chamber into the trachea via a nozzle with a contraction ratio of 1:35, designed with a fifth-order polynomial profile. The nozzle’s exit section, connected to the trachea, was 25 mm long, with an inlet diameter of 12.7 mm and an exit diameter of 17.0 mm. The settling chamber and nozzle were designed following the guidelines provided by Morel [33] and Mehta [34]. The static pressure inside the nozzle, representing the subglottal pressure (Psg), was measured using a Honeywell FPG pressure transducer, with the reported values time-averaged. The airflow was humidified (Hudson RCI, ConchaTherm III) and regulated using a flow controller (Parker, MPC series), a flow meter (MicroMotion Inc, CMF025 Coriolis Flow Meter, Boulder, CO, USA), and a pressure regulator (ControlAir Inc, Type 100 Precision Air Pressure Regulator, Amherst, NH, USA). Polyurethane tubing (3/4 in. OD, 1/8 in. wall) connected the various instruments. Acoustic measurements were captured using a 1/4-inch omnidirectional microphone (Type 4958, Brüel & Kjaer, Nærum, Denmark) placed approximately 30 cm laterally and superiorly to the glottis where it did not interfere with the airflow.

Two-dimensional velocity flow fields were measured using phase-locked planar particle image velocimetry (PIV). The high-speed PIV camera (Phantom Miro 340) was mounted to a Scheimpflug adapter and connected to a Nikon 105 mm lens set to an f-stop of 2.8, with a 12 mm extension tube, achieving a spatial resolution of 124 pixels/mm. Flow illumination was provided by a high repetition rate, dual-cavity Nd laser system (Continuum, Terra PIV). PIV data acquisition and processing were conducted using Davis 10.2 (LaVision).

To minimize laser reflections, fluorescent red dye (Cole-Parmer, Rhodamine WT) was applied to the tissue, and a combination of fluorescent red dye and black paint was used on the silicone models. Additionally, both the ambient air and the glottal flow were seeded with DEHS oil [bis(2-ethylhexyl) sebacate] generated by an atomizer (TSI, model 9306).

Phase-locked intraglottal PIV images were captured at the mid-coronal plane, using a reference signal from electroglottography (EGG) for tissue models and from Psg for synthetic models. The glottal cycle was resolved into 72 phases, with each phase representing an average of 20 images. Data was collected at both low and high Psg values for all models, resulting in six measurements for the human larynx model and 12 for the canine and synthetic models. The PIV imaging and data acquisition were synchronized using a shared reference clock with a timing and synchronization module (NI PXIe-6672) equipped with a high-stability reference clock (temperature-compensated crystal oscillator), all controlled via LabVIEW. The sampling rates for the pressure transducer, EGG, and microphone were 44 kHz using a National Instrument data acquisition system (NI, PXIe-6356). Calibration of all equipment was performed according to the specifications provided by each manufacturer. For further details on the experimental setup and intraglottal flow field measurements, please refer to Oren et al. [35].

### 2.3. Experimental Setup for Elasticity Measurements

**Figure 2:**
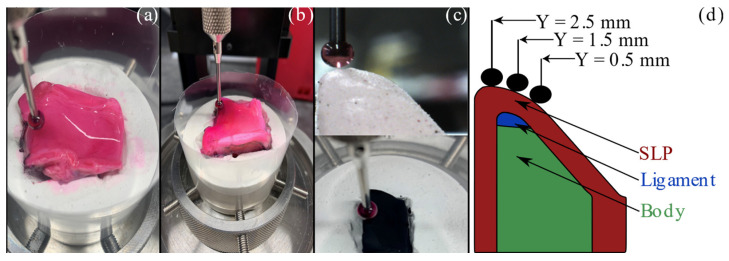
Force–displacement measurements. (**a**) Cadaveric human vocal fold. (**b**) Excised canine vocal fold. The pink color is from the dye applied to minimize the reflections from tissue during PIV measurements (**c**) Synthetic vocal fold before (**top**) and after (**bottom**) applying paint. (**d**) Schematic of the experimental setup used to collect normal force–displacement measurements.

The elastic properties of the vocal folds for the three models were collected according to the procedure outlined by Dion et al. [36]. Following the aerodynamic measurements, the tissue models were bisected in the sagittal plane to create a hemilarynx configuration. A specific holder was molded from plaster material for each larynx (Figure 2). The hemilarynx was affixed to the model mold using cyanoacrylate adhesive. The tissues were kept moist before and during the testing using a phosphate buffered saline (PBS) solution. A similar plaster holder was cast for the synthetic fold, but the saline solution was not used during the testing.

Indentation testing was performed using a mechanical tester (Mach-1, Biomomentum Inc., Laval, QC, Canada) with a 150 g single-axis load cell and 7.5 mg resolution. The force–displacement data were also collected along the mid-coronal plane at the superior, middle, and inferior aspects’ locations: Y = 2.5, 1.5, and 0.5 mm, respectively (Figure 2d). The slope of the surface of each vocal fold was determined by probing the spherical indenter in four locations around the data collection spot to identify the gradient of the surface. The surface was indented 2.0 mm in the normal direction using a 2.5 mm radius spherical indenter. The indenter probe moved at a speed of 0.2 mm/s as the load cell data was sampled at 1.0 kHz. The probe was held for 1.0 s at the maximum displacement before unloading at the same speed of 0.2 mm/s.

The stress was calculated using the equation:

(1) $$\sigma =\frac{F}{A_{\mathrm{contact}}}$$

where F is the force measurement from the load cell and A_contact_ is the contact area between the tissue and the spherical probe. The contact area was determined using the spherical cap area equation:

(2) $$A_{\mathrm{contact}}=2\pi \mathrm{rh}$$

where r is the radius of the spherical indenter and h is the indentation depth [37]. The strain was calculated using the Green’s strain equation:

(3) $$\varepsilon_{G}=\frac{\left(L_{o}^{2}-L^{2}\right)}{2L_{o}^{2}}$$

where L_o_ and L are the undeformed (compressed) and deformed widths, respectively. Young’s modulus (i.e., stiffness) was determined from the derivative of the stress–strain curve:

(4) $$E_{\mathrm{normal}}=\frac{\partial \sigma }{\partial \varepsilon_{G}}$$

## 3. Results

### 3.1. Folds’ Displacement and Glottal Geometry Characteristics

**Figure 3:**
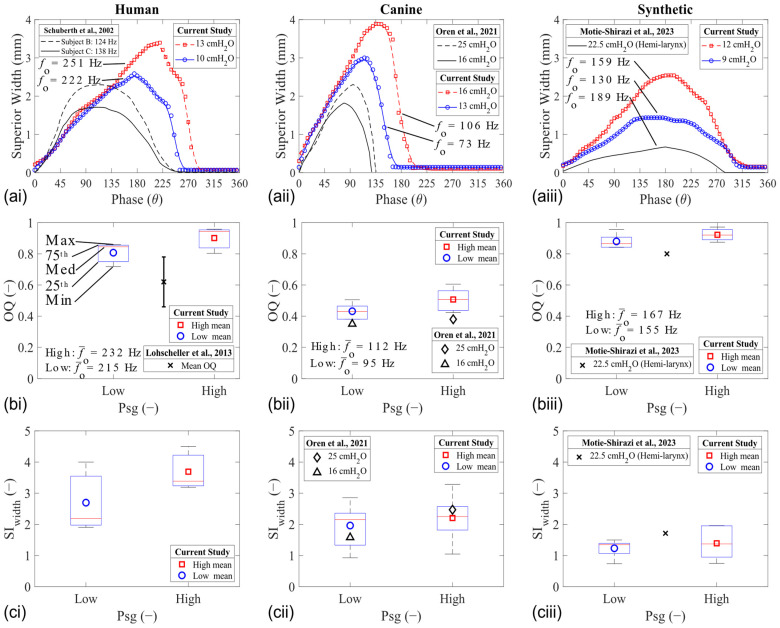
Mid-coronal width analysis of human (**left column**), canine (**middle column**), and synthetic (**right column**) vocal fold models during phonation. (**a**) Glottal width waveform. (**b**) Open quotient. (**c**) Superior width skewness index [38,39,40,41].

Plotting the width of the glottal opening in the mid-coronal shows a comparison between the motion of the folds during vibration in each larynx model. This width was extracted at the superior aspect from the PIV images, and its value is assumed to be proportional to the glottal opening area. All models were characterized by opening, closing, and closed phases during their vibration cycle (Figure 3a). The resultant glottal width waveform was used to calculate the open quotient (OQ) and area skewness index (SI_width_) values (Figure 3b,c). Waveform examples for HL1, CL1, and SL1 are shown in Figure 3a.

The OQ increased with the Psg in all models of the human larynx (Figure 3a). This observation is shown using boxplots (Figure 3b). It is also known that the OQ will vary with changes in vibration frequency [42]. The OQ values of the human larynges was higher than expected, likely because of their physiological cadaveric state. The OQ for the canine larynges was similar to the range observed in normal speakers (0.3–0.6) [42]. The OQ for the synthetic model was similar to what was observed in other studies [27], which is higher compared with the expected range in the human larynx.

The maximum glottal width value increased with the Psg in all laryngeal models. This increase in the displacement value is because a greater force magnitude pushes the fold in the lateral direction during opening. Interestingly, the phase of the maximum displacement also changes in the tissue models, but it remains constant in the synthetic models. For example, the maximum displacement in HL1 occurs at θ = 175° for the low Psg and θ = 220° for the high Psg (23% difference). Likewise, there is a 20% shift in phase for the maximum glottal width CL1. The phase shift was similar between all the synthetic models, around 10%. This difference is also reflected in the SI_width_ measurements, where a larger change was observed for the tissue models compared to the synthetic model (Figure 3c). The difference likely stems from the difference in the VSG (discussed further in Section 3.3. Elasticity Characteristics), which plays a role in facilitating the closing (and closed) phases of the glottis.

**Figure 4:**
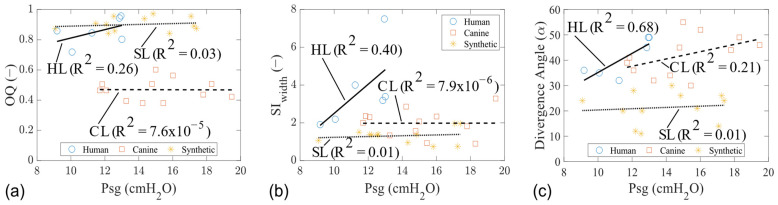
Mid-coronal width trends of human, canine, and synthetic vocal fold models during phonation. (**a**) Open quotient. (**b**) Superior width skewness index. (**c**) Glottal divergence angle at the MFDR.

In all cases, the OQ, SI_width_, and the divergence angle at the instant of the MFDR phase increased with Psg (Figure 4). The SI_width_ values increased the most in the human (Figure 4b), while the canine and synthetic trends remained constant for both the OQ and SI_width_ parameters. The glottal divergence angle, defined as the total angle between the folds at the MFDR phase, was extracted from the PIV measurements (shown in Section 3.2. Glottal Flow Characteristics). The magnitude of the divergence angle at the MFDR phase appeared to be similar in the tissue models and higher than in the synthetic models (Figure 4c).

### 3.2. Glottal Flow Characteristics

**Figure 5:**
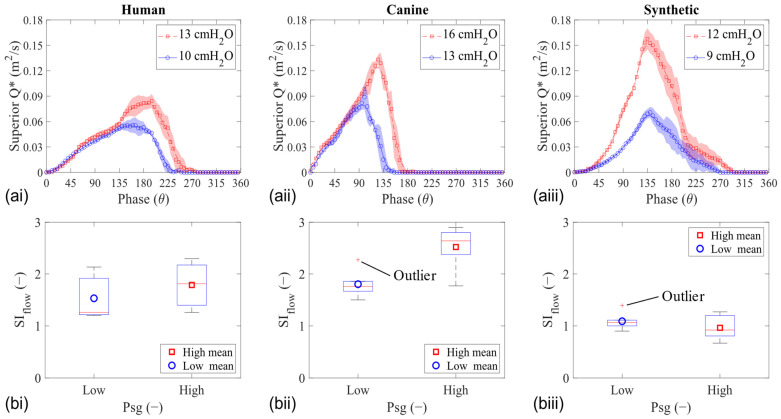
Mid-coronal flow analysis of human (**left column**), canine (**middle column**), and synthetic (**right column**) vocal fold models during phonation. (**a**) Two-dimensional glottal flow waveform. (**b**) Flow skewness index.

Plotting the two-dimensional flow rate (Q*) in the mid-coronal location compares the waveforms from each larynx model. The flow rate was calculated by integrating the axial velocity along the glottal exit (superior edge) at each phase, and examples of the resulting waveforms are shown for HL1, CL1, and SL1 in Figure 5a. Similar to the fold’s displacement, the peak Q* increased with the increasing Psg in all models, but the shift in Q* skewing was observed only in the tissue models. Furthermore, the value of the flow skewness index (SI_flow_) in some of the synthetic models was less than one (SI_flow_ < 1), signifying a prolonged closing phase (compared with opening), which was the opposite of the tissue models (Figure 5b).

**Figure 6:**
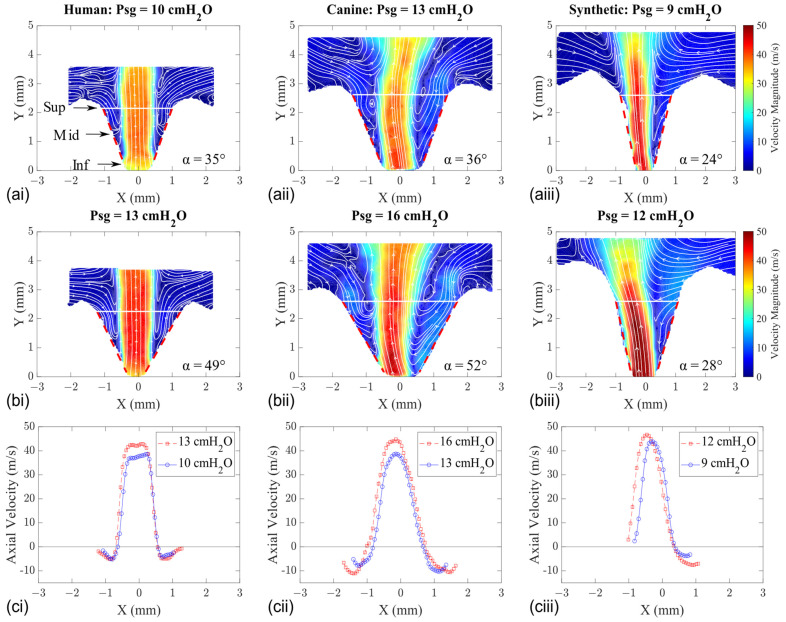
Mid-coronal velocity flow fields of human (**left column**), canine (**middle column**), and synthetic (**right column**) vocal fold models at the MFDR. (**a**) Low Psg. (**b**) High Psg. (**c**) Axial velocity profiles extracted along the superior edge (white horizontal line).

The MFDR was calculated from the derivative of Q*, and the corresponding phase locked average velocity fields for the intraglottal flow in the same larynges as above (HL1, CL1, and SL1) are shown in Figure 6a,b. The total divergence angle between the folds was extracted from each velocity field (marked with dashed red lines along the glottal wall) and is listed in the lower right corner. Axial velocity profiles were also extracted at the glottal exit (the approximate location is marked with a solid white line) and are shown along the bottom row (Figure 6c).

The velocity flow field and profile at the MFDR phase for HL1 are shown in the left column and display glottal flow separation from the medial walls (Figure 6—left column). In both the low and high Psg cases, a stationary vortex is observed along the sides of the intraglottal flow in the CL1 model (Figure 6—middle column). The corresponding axial velocity profiles at the glottal exit are characterized by flow entrainment on both sides (shown as negative velocity values) where flow separation occurs. When intraglottal flow separation occurred in the synthetic models, the glottal jet systematically attached to one of the sides regardless of the Psg (Figure 6—right column). This difference in the intraglottal flow behavior during the phase of the MFDR between the tissue and synthetic models is likely due to the difference in the magnitudes of the divergence angle that forms between the folds. Although the magnitude of this angle increased with the increasing Psg in all models, its values were much greater in the tissue models.

**Figure 7:**
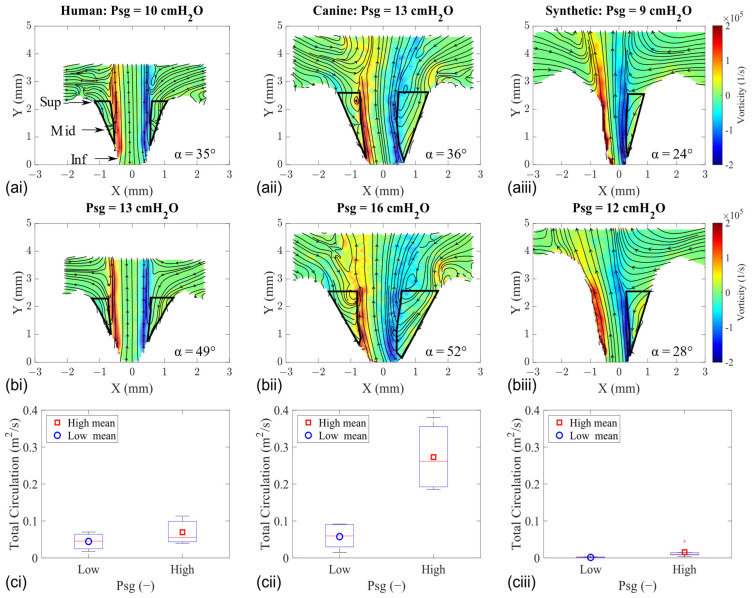
Mid-coronal vorticity flow fields of human (**left column**), canine (**middle column**), and synthetic (**right column**) vocal fold models at the MFDR. (**a**) Low Psg. (**b**) High Psg. (**c**) Total circulation (within the outlined region).

Our investigation into the intraglottal low pressure zones during the MFDR phase involved calculating the flow vorticity (Equation (5)) and circulation (Equation (6)) (Figure 7).

(5) $$\omega =\left(\frac{\partial v}{\partial x}-\frac{\partial u}{\partial y}\right)$$

(6) $$\Gamma =\int^{\;}\omega \partial A$$

where ω is vorticity, u and v are the velocities in the x and y directions, respectively, Γ is circulation, and A is area.

The in-plane vorticity values were determined by taking the curl of the velocity field, and its corresponding fields for HL1, CL1, and SL1 are shown in Figure 7a,b. The circulation in the flow was computed by integrating the vorticity field inside the regions enclosed from the jet’s shear layer to the medial glottal wall. These integration regions are depicted with the solid black line in the vorticity fields, and the data statistics are shown using a boxplot (Figure 7c).

The tissue models exhibit higher levels of circulation than the synthetic model due to higher amounts of entrained flow into the glottis. Lower divergence angles are observed in the synthetic model, and the magnitude of those values does not change much when Psg is increased. Accordingly, the intraglottal circulation remains low. The observed difference in circulation values is also because the intraglottal flow was typically attached to one of the walls in the synthetic models due to the lower divergence angle that does not allow for much of a flow separation region. This resulted in just one integration region (compared with two regions in the tissue models).

**Figure 8:**
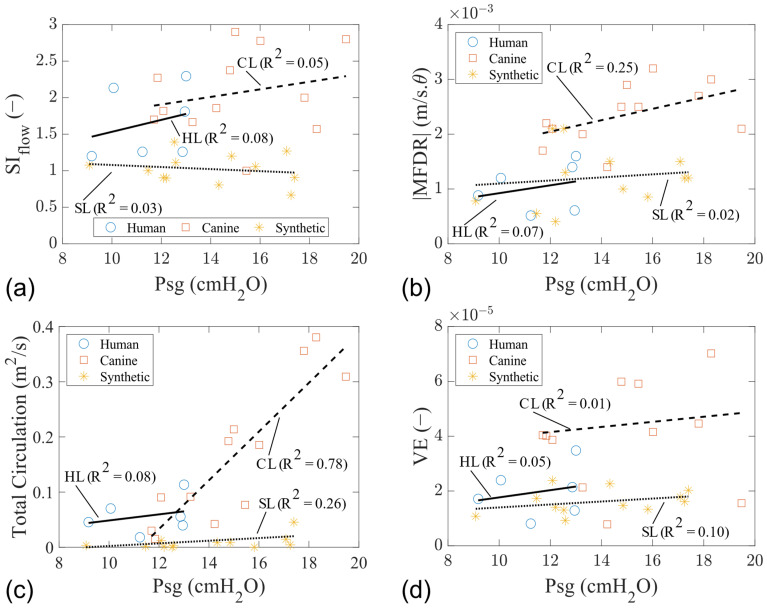
Glottal flow and acoustic characteristics of human, canine, and synthetic vocal fold models. (**a**) Flow skewness index. (**b**) |MFDR|. (**c**) Total circulation. (**d**) VE.

Overall, the parameters of SI_flow_, MFDR, total circulation, and VE increased with the Psg (Figure 8). VE is the radiated acoustic power to aerodynamic power ratio and was calculated using the equation:

(7) $$\mathrm{VE}=\frac{2{\pi R}^{2}10^{-12}10^{\mathrm{SPL}/10}}{\mathrm{PsgQ}}$$

where R is the distance from the microphone to the sound source, SPL is the sound pressure level, Q is the mean glottal flow rate recorded from the upstream flow meter, and Psg is the mean subglottal pressure [43]. These parameters in the canine models are higher than in the human and synthetic models.

### 3.3. Elasticity Characteristics

**Figure 9:**
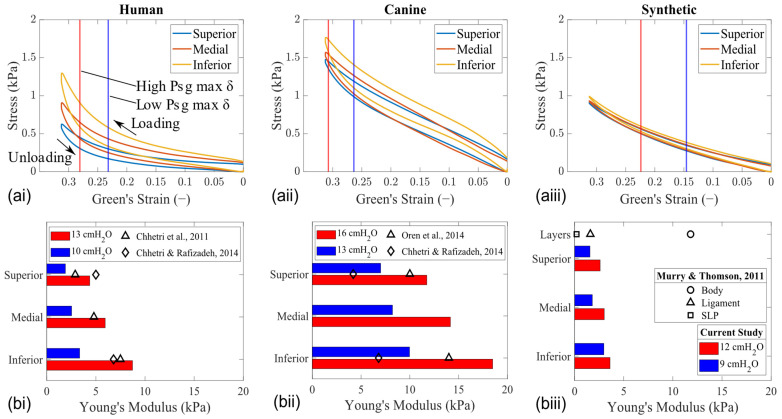
Elasticity measurements along the vertical height of each model in the mid–coronal plane (HL1 [**left column**], CL1 [**middle column**], and SL1 [**right column**]). (**a**) Stress–strain curve. Vertical lines mark the location where the maximum displacement of the folds was observed at high and low Psg values. (**b**) Corresponding Young’s modulus values calculated at the maximum fold displacement [18,19,20,26].

Indentation testing revealed differences in stress–strain profiles among the tissue models and synthetic vocal fold models (Figure 9a). The vertical lines mark the actual length that the fold was displaced (i.e., half of the maximum glottal width, c.f., Figure 3a). Hence, the maximum indentation depth applied to the human and synthetic models was greater than what was observed during vibration. Notably, excised tissue models exhibited hysteresis, indicating energy dissipation between the loading and unloading cycles. Due to the viscoelastic properties of the tissue models, which combine viscous fluidity and elastic solidity, they exhibit both viscous and elastic behavior, as evidenced by the hysteresis in their stress relaxation behavior. In contrast, the synthetic model, made of silicone, is primarily elastic and returns to its original shape after deformation by an external force, resulting in minimal hysteresis.

To ensure that our results are consistent with what has been reported in the literature on synthetic vocal folds models, we performed elasticity tests with and without the mix of fluorescent red dye and black paint that was required for PIV measurements. This confirmed that applying this coating to the synthetic model had a negligible effect on the overall stiffness of the silicone material, as the unloading curves remained nearly identical. The mean differences in Young’s modulus across the superior, medial, and inferior locations before and after painting were 10% and 8% for the low and high Psg displacements, respectively. Therefore, the values reported for the synthetic vocal folds are with respect to no paint.

A significant difference in the VSG was also observed between the tissue and synthetic models (Figure 9b). These modulus values were calculated from the slopes of the stress–strain curves during unloading at the vertical lines marked to show the actual length that the fold was displaced. A similar VSG characteristic was observed in all larynx models, where the stress in the tissue gradually decreases from inferior to superior for a given strain value. However, the gradient in the tissue model becomes more pronounced for displacements corresponding to a high Psg. The horizontal bar graph shows that, as the Psg is increases, the difference between the Young’s modulus (bars of the same color) also increases. For instance, at a low Psg (i.e., low displacement), the difference in the VSG with respect to the HL1 model was 69% higher for CL1 and 19% lower for SL1. As the Psg increased, CL1 remained 42% higher and SL1 was 93% lower than the vertical stiffness gradient in the HL1 model. Overall, these measurements showed that the tissue elasticity of the canine larynx model was stiffer than that of the human, and both were higher than that of the synthetic model. Equivalent results were reported in previous studies using tissue [18,19,20] and synthetic [26] models.

**Figure 10:**
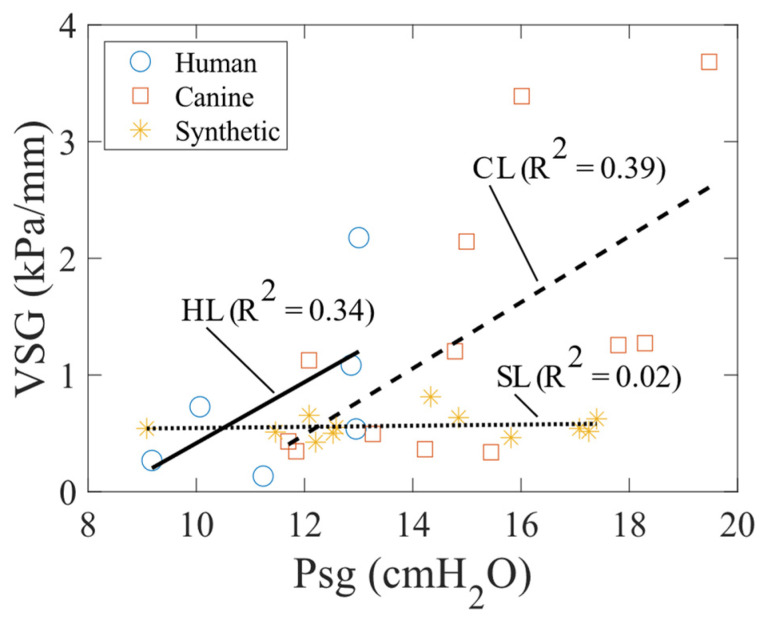
VSG as a function of Psg (i.e., strain) for the human, canine, and synthetic vocal fold models.

The VSG as a function of Psg highlights the notable differences between the tissue and synthetic models (Figure 10). In tissue models, nonlinearity is evident as the VSG increases with strain, due to an increase in Psg. Conversely, in synthetic models, the VSG remains constant with increasing strain, also due to a rising Psg. This behavior occurs because the silicone synthetic model is primarily elastic and exhibits little to no hysteresis.

### 3.4. Summary

Table 1 presents a comparative analysis of mean key vibratory characteristics along with the aerodynamic and elastic properties of the human, canine, and synthetic vocal fold models. The parameters measured include subglottal pressure ($\bar{P}\mathrm{sg}$), fundamental frequency ($\bar{f}o$), flow rate ($\bar{Q}$), open quotient ($\bar{\mathrm{OQ}}$), skewing index of width ($\bar{\mathrm{SI}}$_width_), divergence angle ($\bar{\alpha }$), skewing index of flow ($\bar{\mathrm{SI}}$_flow_), maximum flow declination rate ($\left|\bar{\mathrm{MFDR}}\right|$), circulation at the MFDR phase (MFDR $\bar{\Gamma }$), vocal efficiency ($\bar{\mathrm{VE}}$), and the vertical stiffness gradient ($\bar{\mathrm{VSG}}$).

## 4. Discussion

In this study, we compare, for the first time in the literature, detailed insights into the intraglottal flow field and glottal wall geometry in the mid-coronal planes of both a cadaveric human larynx and a self-oscillating synthetic EPI vocal fold model. The overarching objective of this research was to comprehensively characterize aerodynamic and elastic properties for each model, aiming to provide voice researchers with a deeper understanding of the underlying physics governing each laryngeal model. Trendlines revealed correlations between various acoustic, aerodynamic, and stiffness parameters as a function of Psg during phonation. Parameters such as VE, VSG, divergence angle, total circulation, MFDR, SI_width_, SI_flow_, and OQ exhibited varying degrees of sensitivity to changes in Psg across different laryngeal models. Synthetic models had Psg values similar to human models but slightly lower than canine models. Human models showed the highest frequency values, while the canine and synthetic models had higher flow rates. Human and synthetic models had longer open phases during vibration. Canine models exhibited the highest divergence angles, MFDR values, total circulation values, flow skewing indices, and VE. They also had the highest VSG, indicating greater tissue stiffness variation along the height of the vocal fold. Overall, the tissue models highlighted asymmetry in glottal opening and flow waveforms, which correlated with higher levels of circulation and VSG. Conversely, synthetic laryngeal models composed of silicone exhibit glottal skewing towards opening with a very low VSG, yielding low divergence angles and intraglottal flow circulation.

The results from the current study show that characteristics related to the fold’s displacement and the glottal geometry are similar across the tissue models. Overall, there was a correlation in the models between Psg and the OQ, as well as the divergence angle at the MFDR. The same was observed for the glottal flow characteristics, except for the SI_flow_ and the rotational characteristics in the synthetic vocal fold model. In the tissue models, the SI_flow_ value was larger than one and increased with Psg, indicating that the opening phase’s duration was always longer, and the closing phase’s duration decreased as Psg increased. The opposite was observed for the synthetic vocal folds—the duration of the opening phase decreased with Psg and was usually the same or shorter than the closing phase. Similarly, the tissue models showed higher circulation than the synthetic model due to the higher amounts of entrained air into the glottis and stationary vortices near the superior aspect of the folds.

The canine larynx model presents a faithful morphological and functional approximation to human phonation, albeit with size and stiffness differences. The current study showed that the maximum displacement and OQ values in canine models were within the range observed in normal human speakers, confirming their validity as a model for studying phonatory processes. However, being 40% larger and requiring higher phonation pressures due to the closer proximity of the conus elasticus to the surface of the canine vocal fold necessitate adjustments to the experimental parameters, potentially complicating the direct comparison to human phonation. As with the human larynx data, the repeatability of the results can be a challenge.

The data from the synthetic vocal folds highlight the model’s suitability for controlled, repeatable experiments. Sustaining vibrations for extended periods and manipulating material properties and geometries provide significant experimental flexibility. The current study showed that, in addition to glottal displacement and geometry, a detailed investigation of flow characteristics, such as the SI and MFDR, can be performed to assess the impact of varying Psg values on phonatory dynamics. However, the synthetic models exhibited different intraglottal flow behaviors, such as the persistent attachment to one vocal fold and lower circulation values. Additionally, silicone material properties did not fully replicate the viscoelastic nature of biological tissues. Parameters such as VSG are associated with greater divergence angles, increased circulation, higher values of the MFDR, and glottal skewing towards closing, which were observed in the tissue models. The lack of crucial features, like the VSG, an anterior commissure, and the symmetry of the vocal folds, further limits the synthetic model’s ability to fully simulate realistic vocal fold behavior.

The cadaveric human larynx model can offer the most realistic representation, but its data can be affected by variations in tissue condition. Additionally, the ethical/logistical challenges of obtaining samples can impact the consistency and availability of data. Postmortem changes, such as atrophy, were evident as they affected the elastic properties, OQ, and the maximum glottal width during phonation. Observations like this limit the ability to generalize findings to living conditions.

The comparative analysis underscores the importance of selecting an appropriate model based on the specific research question and experimental requirements. While human cadaveric larynges provide the most accurate physiological representation when fresh, canine larynges and synthetic models offer practical advantages in terms of availability and experimental control. Advancements in synthetic model development help to bridge the gap between biological fidelity and experimental versatility, paving the way for more comprehensive studies of phonation mechanisms.

Over the past decade, there has been a notable surge in research attention directed towards understanding various aspects of glottal dynamics, including aerodynamics, tissue biomechanics, vibrational patterns, and acoustics within the glottis—the space between the vibrating vocal folds. This increased focus can be attributed, in part, to advancements in non-intrusive laser diagnostics and methodological practices, which have facilitated the observation of intraglottal airflow and glottal wall geometry during phonation, especially in excised tissue models.

Advancements in the design and fabrication of synthetic vocal fold models have provided researchers with a powerful tool for investigating the intricate mechanisms of fluid–structure–acoustic interactions, primarily in voice research. A recent breakthrough involves the utilization of three-dimensional printing technology to create these synthetic vocal fold models [44,45], a significant improvement over the traditional casting process as shown in these studies. Studies have emphasized the importance of a VSG in mimicking physiological phonation characteristics, prompting current advancements to integrate this gradient using specialized additive manufacturing techniques [46].

As highlighted earlier, synthetic vocal fold models offer the flexibility to manipulate material properties and geometries to explore their effects, particularly in understanding various voice disorders. By incorporating the VSG, future investigations hold promise for enhancing insights into specific voice disorders, thereby aiding researchers, clinicians, and physicians in refining surgical interventions and treatment outcomes.

Nevertheless, it is worth noting that current synthetic vocal fold models are symmetric along the sagittal and coronal planes and often lack a connection point at one end, such as the anterior commissure. This connection point plays a pivotal role in the overall fluid–structure–acoustic interaction during phonation. Therefore, as a recommendation for future research, efforts should focus on incorporating a connection point at the end of the vocal folds and utilizing the new synthetic vocal fold model developed by Young et al. [46], which incorporates a VSG, to better simulate real-world phonatory conditions.

A limitation of this study is that the normal indentation measurements performed on the tissues may not fully capture the overall biomechanical properties during vibration. Biological tissues, such as laryngeal tissue, exhibit anisotropic behavior, meaning their mechanical properties vary depending on the direction of measurement. This anisotropy results from the complex, fibrous structure of the tissues, where components like collagen fibers and elastin are aligned in specific directions. Consequently, the elasticity of vocal fold tissue differs along various axes, leading to complex mechanical behavior.

In contrast, the synthetic models used in this study, composed primarily of silicone, are close to isotropic, meaning their mechanical properties are similar in all directions. While this uniformity simplifies the modeling process and ensures consistent behavior under applied forces, it does not fully replicate the anisotropic characteristics of real tissue. As a result, the synthetic models may not capture the directional dependence of tissue properties accurately, which could limit their effectiveness in modeling certain aspects of vocal fold elasticity during vibration.

The implications of these differences are particularly relevant when considering the elastic and aerodynamic properties of the models. While silicone makes it suitable for controlled, repeatable experiments, it may not adequately simulate the complex, directional-dependent behavior of biological tissue, particularly in studies that aim to replicate the dynamic interactions within the glottis during vibration. Future studies should consider incorporating more advanced materials or composites that better mimic tissue anisotropy to enhance the fidelity of synthetic models in replicating human vocal fold behavior.

## 5. Conclusions

The experimental investigation provided comprehensive insights into the aerodynamic and biomechanical characteristics of cadaveric human, excised canine, and synthetic vocal fold models during phonation. The methodology employed careful preparations and setups to ensure accurate data collection across all models. Aerodynamic measurements revealed distinct patterns in the glottal width, flow, velocity fields, vorticity, and circulation, highlighting the influence of Psg on phonatory behavior. Biomechanical analysis showcased differences in tissue stiffness and behavior under varying pressures, emphasizing the importance of a VSG for realistic intraglottal airflow. Statistical trends demonstrated correlations between aerodynamic parameters, the vocal folds’ VSG, and phonatory features, underscoring the interplay between aerodynamics and biomechanics in vocal fold function. By presenting these findings, we aim to assist researchers in making informed decisions regarding which model is the most suitable for their study. In summary, the results provide valuable insights into the complex interplay between aerodynamic and biomechanical factors influencing intraglottal airflow and vocal fold vibration across different laryngeal models.

## Figures and Tables

**Table 1:** Summary of mean measured parameters and standard deviation for each vocal fold model.

Model	$\bar{P}sg$ (cmH_2_O)	$\bar{f}o$ (Hz)	$\bar{Q}$ (LPM)	$\bar{OQ}$ (−)	${\bar{SI}}_{\mathrm{width}}$ (−)	$\bar{\alpha }$ (Deg.)	$\bar{SI}$ _flow_(−)	$\left|\bar{MFDR}\right|$ (m/s·θ)	$MFDR\;\bar{\Gamma }$ (m^2^/s)	$\bar{VE}$ (−)	$\bar{VSG}$ (kPa/mm)
Human	10.26 ± 1.03	215.67 ± 75.70	15.93 ± 8.80	0.81 ± 0.08	2.70 ± 1.14	37.00 ± 5.57	1.53 ± 0.52	8.66 × 10^−4^ ± 3.43 × 10^−4^	4.46 × 10^−2^ ± 2.62 × 10^−2^	1.64 × 10^−5^ ± 7.95 × 10^−6^	0.34 ± 0.27
	12.94 ± 0.07	232.00 ± 79.23	24.48 ± 12.87	0.90 ± 0.09	3.69 ± 2.43	48.33 ± 3.06	1.79 ± 0.52	1.20 × 10^−3^ ± 5.24 × 10^−4^	6.95 × 10^−2^ ± 3.88 × 10^−2^	2.30 × 10^−5^ ± 1.10 × 10^−5^	0.90 ± 0.63
Canine	13.23 ± 1.31	94.33 ± 25.91	24.16 ± 6.18	0.45 ± 0.45	1.96 ± 0.65	35.33 ± 3.82	1.80 ± 0.24	1.98 × 10^−3^ ± 3.53 × 10^−4^	5.77 × 10^−2^ ± 3.00 × 10^−2^	3.68 × 10^−5^ ± 1.72 × 10^−5^	0.49 ± 0.28
	17.02 ± 1.70	111.67 ± 25.01	31.24 ± 5.91	0.49 ± 0.49	2.20 ± 0.73	48.50 ± 3.95	2.52 ± 0.38	2.73 × 10^−3^ ± 3.59 × 10^−4^	2.73 × 10^−1^ ± 7.89 × 10^−2^	5.40 × 10^−5^ ± 2.41 × 10^−5^	2.13 ± 1.05
Synthetic	12.40 ± 1.80	155.67 ± 25.55	29.93 ± 8.26	0.87 ± 0.78	1.23 ± 0.26	19.00 ± 5.80	1.09 ± 0.15	9.99 × 10^−4^ ± 5.66 × 10^−4^	1.46 × 10^−3^ ± 1.16 × 10^−3^	1.30 × 10^−5^ ± 2.54 × 10^−6^	0.45 ± 0.14
	15.48 ± 1.97	167.17 ± 17.10	39.85 ± 9.76	0.92 ± 0.82	1.39 ± 0.46	24.67 ± 5.12	0.96 ± 0.21	1.45 × 10^−3^ ± 3.20 × 10^−4^	1.55 × 10^−2^ ± 1.37 × 10^−2^	1.92 × 10^−5^ ± 3.31 × 10^−6^	0.54 ± 0.20

## Data Availability

All data generated or analyzed during this study are included in this article. Further inquiries can be directed to the corresponding authors.
